# Supplementary material for: Bioinformatics Predicted Linear Epitopes of the Major Coat Protein of the Beet Yellows Virus for Detection of the Virus in the Cell Extract of the Infected Plant
Source: BioTech (Basel). 2022 Nov 10;11(4):52. doi: 10.3390/biotech11040052 (PMC9680324; doi:10.3390/biotech11040052)
Supplement: Supplementary file 1 [file biotech-11-00052-s001.zip › biotech-1991738-supplementary.pdf]

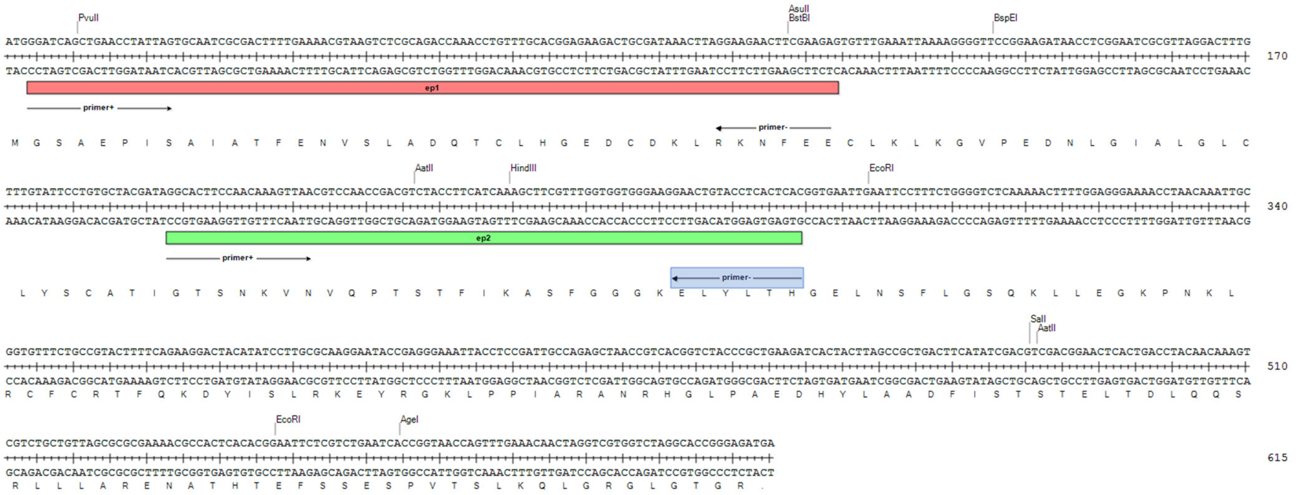

Figure S1. Positions of epitopes and primers on the BYV CP sequence. Open reading frame of the beet yellows virus protein p22 and its amino acid sequence. The red box is epitope 1 and the green box is epitope 2, and black arrows indicate primers without restriction sites used to amplify these epitopes. *Escherichia coli* rare codons are: AGG, AGA, CTA, ATA, CGA and CCC. To optimize codons for strong protein expression, we excluded rare codons from our primers. There is one rare codon in epitope 1 (AGG-R) but it located out of the primer sequence.
